# Phylogeny, envenomation syndrome, and membrane permeabilising venom produced by Australia’s electric caterpillar *Comana monomorpha*

**DOI:** 10.1038/s41598-024-65078-1

**Published:** 2024-06-19

**Authors:** Mohaddeseh H. Goudarzi, Samuel D. Robinson, Fernanda C. Cardoso, Michela L. Mitchell, Lyn G. Cook, Glenn F. King, Andrew A. Walker

**Affiliations:** 1 Institute for Molecular Bioscience, The University of Queensland, St Lucia, QLD 4072 Australia; 2 Australian Research Council Centre of Excellence for Innovations in Protein and Peptide Science, Institute for Molecular Bioscience, The University of Queensland, Brisbane, QLD 4072 Australia; 3 Department of Toxinology, Women’s and Children’s Health Network, North Adelaide, SA 5006 Australia; 4 School of the Environment, The University of Queensland, St Lucia, QLD 4072 Australia

**Keywords:** Aerolysin, Venom toxin, Pore-forming protein, Zygaenoidea, Cytolysis, DNA barcoding, Proteomics, Molecular evolution

## Abstract

Zygaenoidea is a superfamily of lepidopterans containing many venomous species, including the Limacodidae (nettle caterpillars) and Megalopygidae (asp caterpillars). Venom proteomes have been recently documented for several species from each of these families, but further data are required to understand the evolution of venom in Zygaenoidea. In this study, we examined the ‘electric’ caterpillar from North-Eastern Australia, a limacodid caterpillar densely covered in venomous spines. We used DNA barcoding to identify this caterpillar as the larva of the moth *Comana monomorpha* (Turner, 1904). We report the clinical symptoms of *C. monomorpha* envenomation, which include acute pain, and erythema and oedema lasting for more than a week. Combining transcriptomics of venom spines with proteomics of venom harvested from the spine tips revealed a venom markedly different in composition from previously examined limacodid venoms that are rich in peptides. In contrast, the venom of *C. monomorpha* is rich in aerolysin-like proteins similar to those found in venoms of asp caterpillars (Megalopygidae). Consistent with this composition, the venom potently permeabilises sensory neurons and human neuroblastoma cells. This study highlights the diversity of venom composition in Limacodidae.

## Introduction

Larval lepidopterans (caterpillars) are specialised for feeding and growth whereas adults (moths or butterflies) are specialised for dispersal and mating. Their exposure at feeding sites and inability to move rapidly make caterpillars vulnerable to predation. As a consequence, numerous defence mechanisms have evolved in caterpillars, including camouflage, irritative hairs, and venom-injecting spines^1^. Envenomation by certain caterpillar species can have significant effects on humans. For example, envenomation by *Lonomia* spp. (Saturniidae) causes a haemorrhagic envenomation syndrome which can result in death^2^. However, aside from species of *Lonomia*, the venoms of other venomous lepidopterans remain underexplored at the molecular level.

To address this knowledge gap, we recently characterised the venom of the north American saddleback caterpillar *Acharia stimulea* (Zygaenoidea: Limacodidae)^3^ and found its composition to be very similar to that of the Australian limacodid, *Doratifera vulnerans*^4^. The venom of both species was dominated by peptides. In each case, three families of peptides were particularly well represented: adipokinetic hormone/corazonin-related neuropeptides (ACPs), cecropin-like peptides, and inhibitor cystine knot (ICK) peptides^3,4^. In striking contrast, representatives of another zygaenoid family, the asp or puss caterpillars (Megalopygidae), produce venoms that lack these peptides but are rich in aerolysin-like pore-forming proteins (PFPs) and other families of linear and disulfide-rich peptides^5^. Further studies are required to distinguish if the venoms of Limacodidae and Megalopygidae evolved independently^6^ or were mutually inherited from a shared venomous ancestor^7^.

In this study, we analysed the venom composition of a limacodid species from north-eastern Australia known locally as the ‘electric’ caterpillar. Commonly occurring on lillypilly (*Syzygium* spp.), this caterpillar is regarded as a public menace by many gardeners, outdoorspeople, and medical practitioners, and is a frequent cause of presentation at medical clinics (Renae Vagg, Magnetic Island Health Service, Qld, Australia, personal communication). The two limacodid species for which venoms have previously been characterised, *A. stimulea* and *D. vulnerans*, have venom spines clustered at both the anterior and posterior, and sometimes the lateral margins, of the caterpillar. The electric caterpillar is morphologically distinct, being covered with spines on most of its dorsal and lateral surfaces.

In this study, we used DNA barcoding to identify the electric caterpillar as the larva of the limacodid moth *Comana monomorpha* (Turner, 1904) and present an envenomation case study. We present a detailed analysis of the venom composition and activity, revealing a venom that more closely resembles that of previously characterised megalopygid rather than limacodid caterpillars, consistent with the observed envenomation syndrome.

## Methods

### Insect collection and venom harvest

Caterpillars were collected on private land in Townsville, Queensland, Australia with permission of the landowners. They were maintained in the laboratory at 23 °C with a 12 h:12 h light/dark cycle on a bouquet of small lillypilly branches with the stems immersed in water. For RNA-Seq, venom scoli were cut from eight last-instar caterpillars of unknown sex and stored in RNAlater (Thermo Fisher, Waltham, MA, USA) at − 80 °C. The envenomation case study resulted from envenomation by a caterpillar of unknown sex.

Venom was harvested using parafilm suspended on a 20 × 30 mm wire loop with an extended wire handle. The parafilm was gently pressed onto caterpillars, resulting in the deposition of numerous tiny drops of venom from the spines. These drops were recovered by rinsing the parafilm with 20 µL of ultrapure water and transferring the liquid to a 1.5 mL tube. After collecting venom from multiple individual caterpillars into a single tube, the liquid was lyophilised, resuspended in ultrapure water, and the protein concentration estimated using a Nanodrop spectrophotometer (Thermo Fisher) with conversion factor 1 AU/cm^−1^ = 1 mg/mL. Over the course of this study, venom from several caterpillars of each of the final three instars was harvested and pooled for analysis.

### Transcriptomics

To extract RNA, tissue was transferred to 0.5 mL TRIzol reagent (#15-596-018, Invitrogen, Carlsbad, CA, USA) and homogenised with a THq digital homogeniser (Omni International, Kinnesaw, GA, USA) for 30 s, and allowed to stand at room temperature for 5 min. After addition of 100 µL chloroform, the sample was shaken and allowed to stand at room temperature for a further 5 min, then centrifuged at 12,000 *g* at 4 °C for 10 min. The upper aqueous phase containing RNA was removed with a pipette to another RNAse-free 1.5 mL tube. Then, 250 µL of ice-cold 2-propanol was added to precipitate RNA, the sample was vortexed to mix, and then allowed to rest at room temperature for 5 min before centrifugation at 12,000 *g* at 4 °C for 10 min. The supernatant was removed, and 500 µL ice-cold 75% ethanol was added to wash the pellet, followed by another round of centrifugation at 12,000 *g* at 4 °C for 10 min. The supernatant was removed and the pellet was allowed to partially air-dry before resuspending in RNAse-free ultrapure water. RNA concentration (62 ng/µL) and purity (A_260_/A_280_ ratio) were estimated using a Nanodrop spectrophotometer. PolyA^+^ RNA isolation, TruSeq stranded mRNA library construction, and 150 bp insert paired-end nucleotide sequencing on an Illumina NextSeq 500 instrument were performed at the Institute for Molecular Bioscience Sequencing Facility, The University of Queensland (UQ). De novo assembly was performed using an automated pipeline for trimming, correcting, and assembling RNA-Seq reads using fqtrim (https://github.com/gpertea/fqtrim), prinseq^8^ and Trinity 2.4.0^9^ yielding 13,257 contigs. Trinity’s TransDecoder 5.3.0 (https://github.com/TransDecoder/TransDecoder) software was then used to translate open reading frames > 75 bp, resulting in 42,815 open reading frames (ORFs) encoding polypeptides > 25 amino acids in length. These amino acid sequences, together with a list of 200 common mass spectrometry (MS) contaminants, were used as a search database for comparison to mass spectra obtained from venom.

### Collection of clinical data

De-identified data from one person accidentally envenomated by an electric caterpillar was collected on a private residence in Townsville, Queensland, under UQ Human Ethics project 2023/HE000629.

### Phylogenetics

Sequence data deposited by Lin et al.^7^ were downloaded, comprising five loci: cytochrome oxidase subunit 1 (CO1), 28S and 18S RNA, elongation factor 1α, and *wingless*. The *D. vulnerans* sequences were used as queries in BLASTn searches (version 2.11.0) against our transcriptome databases to retrieve the corresponding sequences for *C. monomorpha*, *A. stimulea*, *Doratifera casta*, *Comana albibasis*, and *Thosea* sp., which were trimmed using the *D. vulnerans* sequences as a guide. Concatenated gene sequences were aligned using MAFFT 7.526^10^ with default parameters, and the alignment analysed using IQ-TREE 2.3.4 (www.iqtree.org)^11,12^. IQ-TREE determined the optimal substitution model for this dataset to be GTR + F + I + R5. 1000 bootstraps were performed. A partitioned model in which the models for the five loci listed above were respectively GTR + F + I + G4, SYM + I + G4, K2P + I + G4, GTR + F + I + G4, and TVMe + I + G4, and a tree constructed using Bayesian estimation of phylogeny in MrBayes 3.2.7 software (https://nbisweden.github.io/MrBayes/download.html)^13^ (based on five million cycles with two cold and two hot chains, a burn-in of 37,500 cycles, and the mixed models setting) produced highly similar trees.

### Proteomics

Approximately 50 µg dry weight of whole venom (estimated using A_280_ measured on a Nanodrop spectrophotometer) was used per sample. Reduced, alkylated and trypsinised samples were prepared by diluting the sample to be analysed in reducing/alkylating solution (1% 2-iodoethanol, 0.25% triethylphosphine, 48.75% acetonitrile (ACN), 50 mM ammonium carbonate pH 11.0) and incubating the sample for 1 h at 37 °C. Samples were then dried by vacuum centrifugation and resuspended in digestion reagent (20 ng/µL sequencing-grade trypsin (#7575, Sigma Aldrich, St Louis, MO, USA) in 40 mM ammonium bicarbonate pH 8.0, 10% ACN) before quenching in extraction reagent (50% ACN, 5% formic acid (FA)), vacuum centrifugation, and reconstitution in 1% FA. Reduced and alkylated (but not trypsinised) samples were prepared using the same method but skipping the digestion step. Native samples were diluted in 1% FA.

Samples for MS analysis were loaded onto a 150 mm × 0.1 mm Zorbax 300SB-C18 column (Agilent, Santa Clara, CA, USA) on a Shimadzu Nano LC system (Shimadzu, Kyoto, Japan) with the outflow coupled to a SCIEX 5600 Triple TOF (Framingham, MA, USA) mass spectrometer equipped with a Turbo V ion source and incubated at 60 °C. Peptides were eluted over a 70 min gradient of 1–40% solvent B (90% ACN and 0.1% FA) in solvent A (0.1% FA) at a flow rate of 0.2 mL/min. For MS1 scans, *m/z* was set between 350 and 2200. Precursor ions with *m/z* 350–1500 with a charge from + 2 to + 5, and signals with > 100 counts/s (excluding isotopes within 2 Da) were selected for fragmentation, and MS2 scans were collected over a range of 80–1500 m*/z*. Scans were obtained with an accumulation time of 250 ms and a cycle of 4 s. The generated mass spectra were compared to the amino acid database generated from the venom-gland transcriptome of *C. monomorpha* using the Paragon 4.0.0.0 algorithm in ProteinPilot 4.0.8085 software (https://sciex.com/products/software/proteinpilot-software) (SCIEX, Framingham, MA, USA). The open reading frames (ORFs) encoding polypeptides detected in venom were re-analysed by re-mapping the trimmed reads with Geneious Prime 2021.2.2 software (https://www.geneious.com). Final protein annotation was performed using SignalP^14^, BLAST searches^15^ with cutoff E < 1e^−5^ against the UniRef90 and SwissProt databases, and HMMER searches^16^ against the Pfam database. Normalised spectral counts were calculated using the precursor signal data from reduced, alkylated and trypsinised sample. From the ProteinPilot peptide summary, the three tryptic peptides with highest precursor intensity were averaged to give an abundance estimate. Polypeptides were then named according to rational nomenclature guidelines^17^. Selected peptide and protein 3D structures were predicted using AlphaFold 2^18^.

### Gel electrophoresis of the venom

10% SDS-PAGE gels were used to analyse whole venom composition. Gels were electrophoresed at 120 V for 1 h (PowerPacHC, BIORAD). Gels were stained (0.2% Coomassie blue, 10% acetic acid, 40% methanol) on a shaker for two hours and destained (10% acetic acid, 40% methanol) with four 1 h washes. The bands were then cut and prepared for proteomics experiments as explained in Sect. “Proteomics”. Approximately 30 µg dry weight of whole venom (estimated using A_280_ measured on a Nanodrop spectrophotometer) was used per well.

### Calcium imaging of dorsal root ganglion (DRG) cells

Dorsal root ganglion (DRG) cells were acquired from a 4–8-week-old male mouse and plated in Dulbecco’s modified Eagle’s medium (Thermo Fisher) containing 10% fetal bovine (FBS; Assaymatrix, Melbourne, Australia) and penicillin/streptomycin (Gibco) on a 96-well poly-D-lysine–coated culture plate (Corning, Corning, NY, USA) and maintained overnight. Cells were loaded with Fluo-4 AM calcium indicator, based on the manufacturers’ instructions (Thermo Fisher). After 1 h, the dye solution was replaced with assay solution (1 × Hanks’ balanced salt solution and 20 mM HEPES). Fluorescence changes due to fluctuations in intracellular calcium ([Ca^2+^]_*i*_) of approximately 100–150 DRG cells per experiment were monitored in parallel using a Nikon Ti-E Deconvolution inverted microscope, equipped with a Lumencor Spectra LED Light source. Images were obtained using a 20 × objective at 1 frame/s (excitation, 485 nm; emission, 521 nm). For each experiment, baseline fluorescence was monitored for 20 s, and at 30 s, the assay solution was replaced with an assay solution containing whole venom (1:1000 dilution). Experiments were approved by the UQ Animal Ethics Committee (TRI/IMB/093/17).

### High-content fluorescent calcium and nucleic acid assay

Cell culture reagents were from Life Technologies Corporation, CA, USA, unless otherwise stated. The human neuroblastoma cell line SH-SY5Y was cultured in Roswell Park Memorial Institute (RPMI) medium supplemented with 15% FBS and 2 mM L-glutamine. Cells were maintained at 37 °C in a humidified 5% CO_2_ incubator, and sub-cultured every 3–4 days in a ratio of 1:5 using 0.25% trypsin/EDTA. Bioassay reagents were from Sigma-Aldrich, MO, USA, unless otherwise stated. The assay buffer contained (in mM) 140 NaCl, 11.5 glucose, 5.9 KCl, 1.4 MgCl_2_, 1.2 NaH_2_PO_4_, 5 NaHCO_3_, 1.8 CaCl_2_ and 10 HEPES pH 7.4. Peptides were evaluated for membrane permeabilising activity in SH-SY5Y cells using a Fluorescent Imaging Plate Reader (FLIPR^PENTA^; Molecular Devices, CA, USA) as previously described^19,20^. Briefly, SH-SY5Y cells were plated at 40,000 cells per well in 384-well flat clear-bottom black plates (Corning, Corning, NY, USA) and cultured at 37 °C in a humidified 5% CO_2_ incubator for 48 h before commencing assays. Cells were loaded with 20 μL per well of Calcium 4 dye (Molecular Devices) reconstituted in assay buffer containing 50 µM propidium iodide, and plates incubated for 30 min at 37 °C in a humidified 5% CO_2_ incubator. Fluorescence responses were recorded at excitation and emission wavelengths of 470–495 and 515–575 nm, respectively, for intracellular calcium measurements and excitation and emission wavelengths of 470–495 and 565–625 nm, respectively, for DNA exposure measurements. Recordings were made for 10 s to set the baseline, and for 600 s after addition of peptides. Triton X-100 at 0.025% was used as positive membrane permeabilising control.

### Statistical analysis

Statistics for proteomic identification of peptides and proteins were produced by ProteinPilot 4.0.8085 software (SCIEX). EC_50_ values for FLIPR assays were calculated using area under the curve or maximum–minimum fluorescence values fitted to a four-parameter non-linear regression to the data in Prism 10 (GraphPad, La Jolla, CA, USA).

### Ethical approval and consent to participate

All animal experiments were approved by the UQ Animal Ethics Committee (TRI/IMB/093/17). All experiments were performed in accordance with relevant guidelines and regulations. The collection of human clinical data was approved under UQ Human Ethics project 2023/HE000629. The authors confirm that consent for participation in this study was declared by all participants in written records kept at The University of Queensland.

## Results

### Envenomation case study

Anecdotal reports from residents of the Townsville region (Queensland, Australia) suggested that envenomation from the ‘electric caterpillar’ could cause symptoms not usually associated with limacodid envenomations, including long-lasting pain and irritation as well as anaphylaxis-like effects (Supplementary Figure S1). Envenomation by electric caterpillars is a common reason for patients to present at healthcare services in this region. Patients typically report severe itch, sting or burning, and often long-lasting lesions. However, we are not aware of any information in the scientific or medical literature relating specifically to envenomation by this species. For this reason, we here report a case study of electric caterpillar envenomation from a Townsville resident.

**Figure 1:**
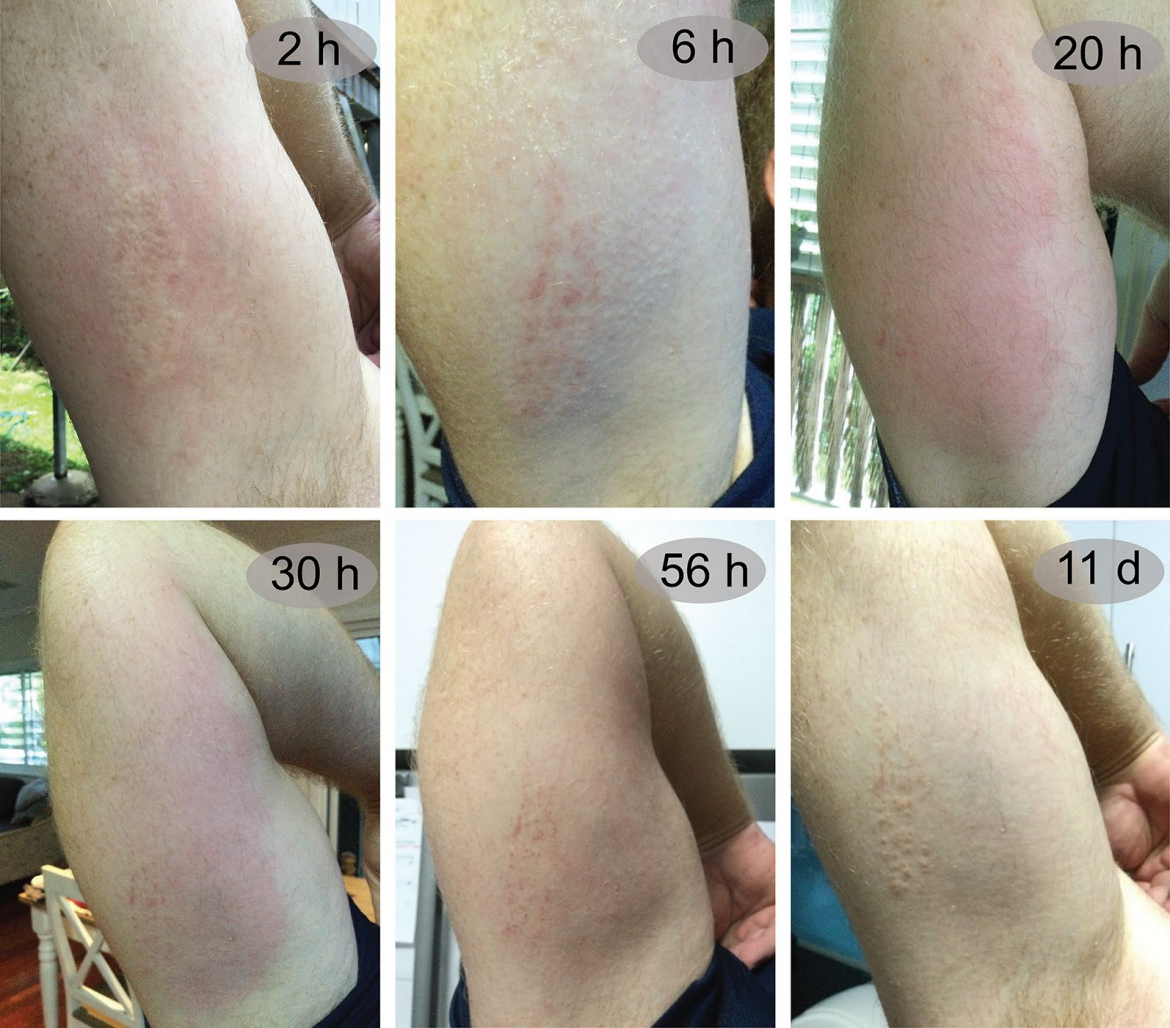
*Comana monomorpha* envenomation. Progression of skin lesion over eleven days in envenomation case study. Photos by M. L. Mitchell.

In this case study (Table 1, Fig. 1), envenomation occurred under the upper arm of an adult male (47 years old) while gardening; this was the first time the patient had been envenomated by this species of caterpillar. Erythema and oedema occurred in a 200 × 50 mm area of skin within 30 min of envenomation. First aid application of ice pack, hot water and Stingose® gel (20% w/v aluminium sulphate hydrate) did not alleviate the symptoms. The pain was described as radiating along the arm short of reaching the fingers, with a mixed numbness and tingling sensation. Pain was still present but not as intense as at 1–2 h post envenomation, and the swelling had almost resolved by ~ 4 h post envenomation.

**Table 1:** Case study of the envenomation syndrome of *Comana monomorpha*.

Time post envenomation	Symptoms
0 h	Mild reaction to initial contact, quickly escalated to severe stinging; casualty reported he “felt like he had been stung by a bee”
0.5 h	Raised welt and stinging
1 h	Raised welt, prickled appearance due to spines, extreme stinging
2 h	Irritability in mood, still extreme stinging
6 h	Toxins still acting on muscles, slightly painful to touch; welt and redness receded and a few localised bumps where spines entered skin; Light bruising evident
8 h	Localised pain and muscles still being affected
18 h	Extreme redness on entire underarm, evidence of grazing where contact was made with caterpillar
3 d	Symptoms and rash absent—some mild residual pain
9 d	Envenomation site became raised and mild itchiness was experienced, no pain present
10 d	Welts still evident, redness entirely reduced, some light bruising still evident

### Species identification and phylogenetics

**Figure 2:**
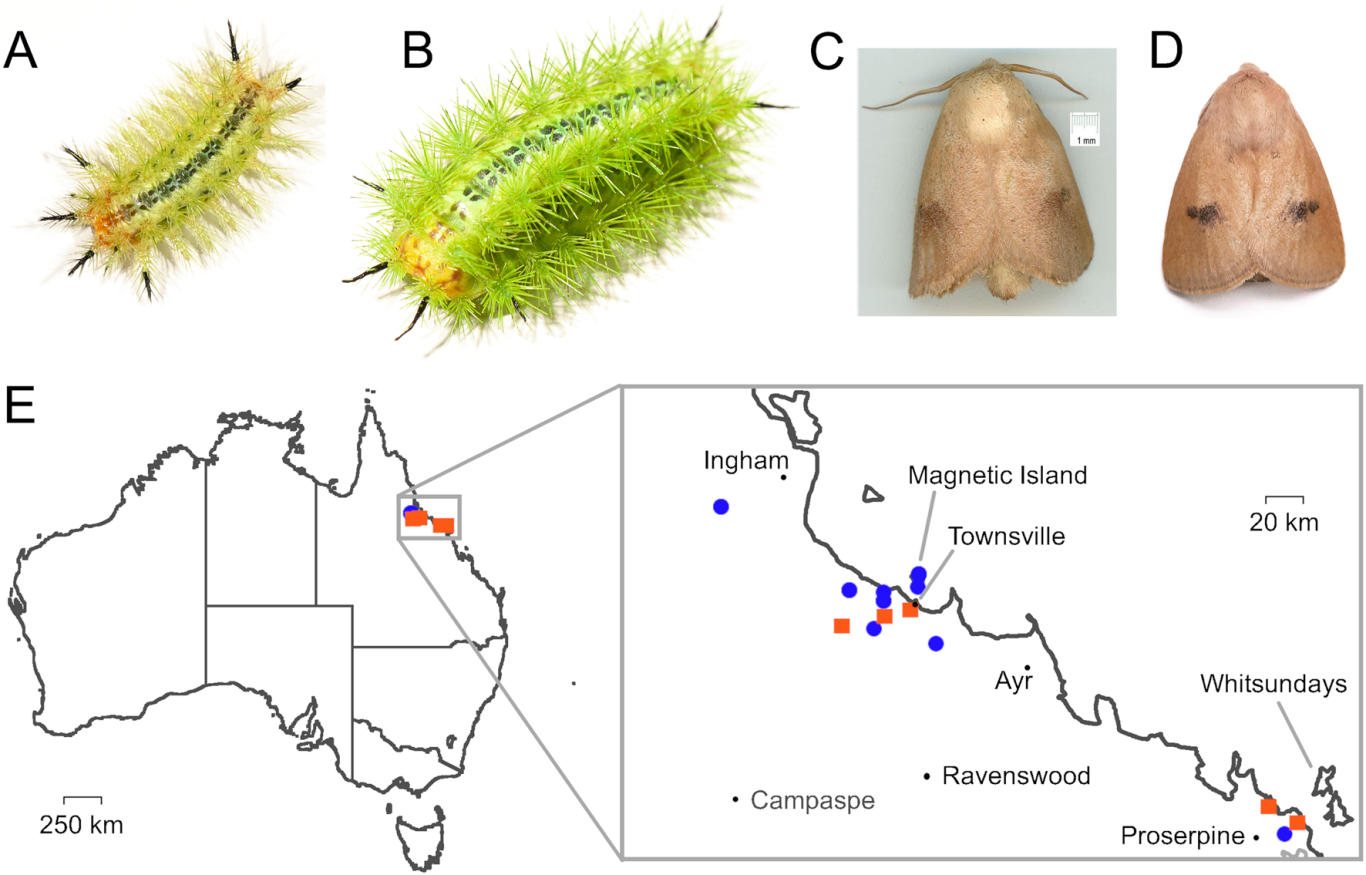
The electric caterpillar, *Comana monomorpha*. (**A**, **B**) Caterpillars of third-final and final instars, respectively. Photos by S. D. Robinson. (**C**) Photos of *C. monomorpha* from the Townsville region that yielded COI sequence of BOLD sample LOQT663-06. Photo by Graeme V. Cocks, reproduced without alteration. (**D**) Moth raised from electric caterpillars in the laboratory in this study. Photo by S. D. Robinson. (**E**) Geographic distribution of iNaturalist records of electric caterpillars (blue circles) and *C. monomorpha* moths (orange squares). Map from vemaps.com.

Electric caterpillars (Fig. 2A,B) have four rows (two lateral and two dorsolateral) of venom scoli on each body segment. The larvae are lime green, varying from a bright yellow-green to a darker emerald green. Along the dorsal midline, a series of dark blue or black rosettes or X-marks form a continuous or discontinuous stripe. Another series of dark blue or black markings, consisting more of a series of circular arcs, runs between the dorsal and dorsolateral scoli. The head, and to a lesser extent the terminalia, may appear yellowish or orange. Four scoli at the anterior end and two at the posterior are coloured black.

To facilitate DNA barcoding and determination of venom composition, we performed RNA-Seq of RNA extracted from the venom scoli of a late instar electric caterpillar. We retrieved the cytochrome oxidase subunit 1 (*CO1*) nucleic acid sequence from our larval venom spine transcriptome using the BLASTn algorithm with the *CO1* sequence of *D. vulnerans* as the query. Submitting the obtained *CO1* gene to the Barcode of Life Data system (BOLD, www.barcodinglife.org)^21^ identification engine resulted in five matches with 99.68–100.00% identity, all to specimens of *Comana monomorpha* identified by curators of the Australian National Insect Collection or the entomologist Graeme V. Cocks. No BOLD database *CO1* entries for *C. monomorpha* had < 99.68% identity to the sequence generated in this study and matches to *CO1* of other species were < 93%.

After the last instar, electric caterpillars spun a dark brown spherical cocoon. Adults emerged several weeks later, with creamy brown colouration and darker spots on the wings, highly similar to the appearance of *C. monomorpha* from which the closely matching *CO1* sequences were obtained (Fig. 2C,D). To examine if the geographic distribution of the electric caterpillar is consistent with this identification, we examined iNaturalist (www.inaturalist.org) records of electric caterpillars and moths *of C. monomorpha*. We found the observations closely agreed to the known distribution, with the range limited to coastal Queensland south of Mission Beach and north of Mackay, with two clusters of observations for both larvae and adults centred around Townsville/Magnetic Island and Proserpine/Airlie Beach (Fig. 2E). These data provide a species level identification for the electric caterpillar as *Comana monomorpha*.

**Figure 3:**
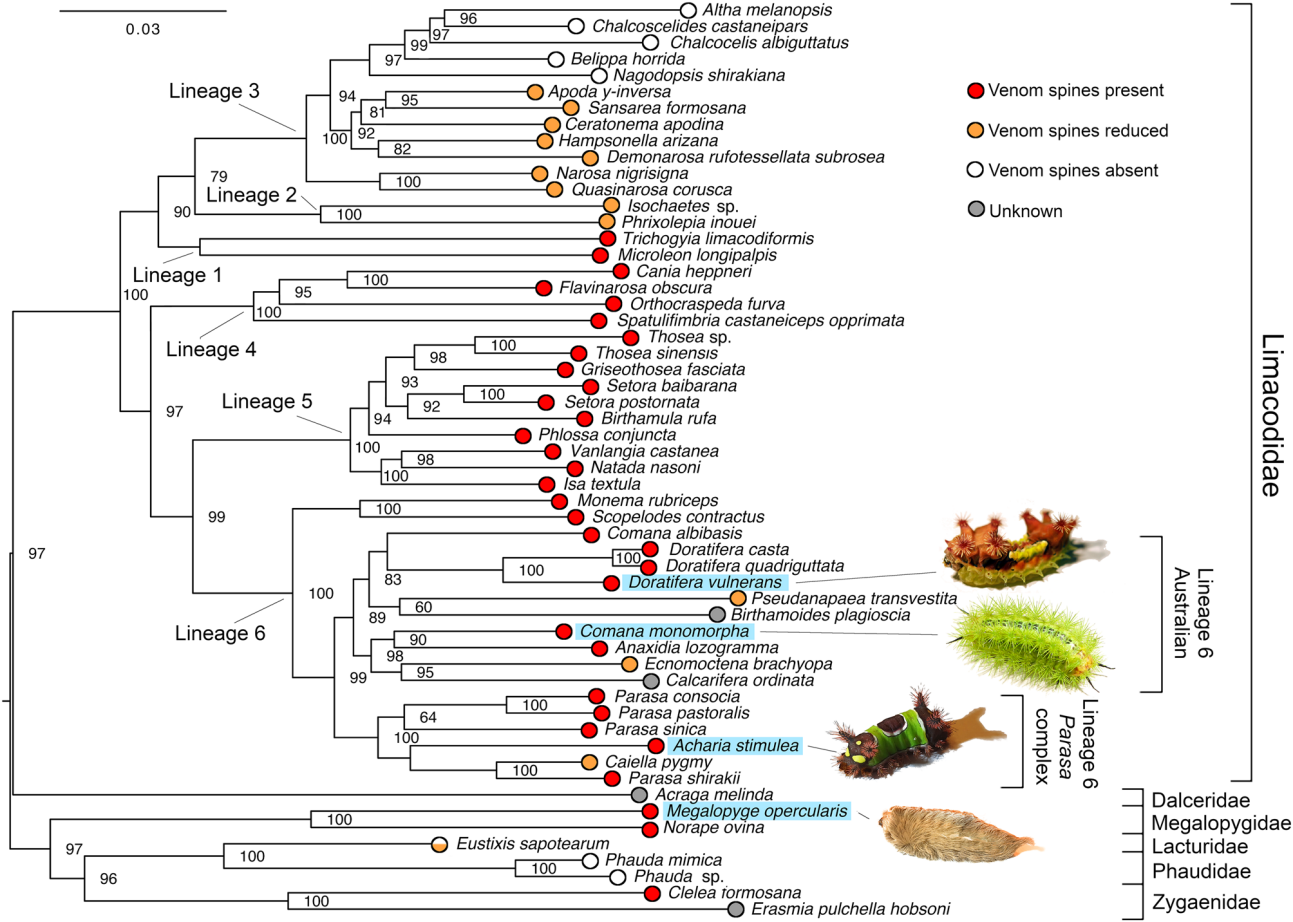
Maximum likelihood phylogeny of Limacodidae. Tree is midpoint-rooted and node labels indicate bootstrap values. The six lineages proposed by Lin et al. ^22^ are labelled. Species with characterised venom compositions are highlighted in blue. Photos by S. D. Robinson (*C. monomorpha*), A. A. Walker (*A. stimulea*, *M. opercularis*), and J. Jin (*D. vulnerans*).

To investigate the phylogenetic position of *C. monomorpha*, we downloaded the sequences of five loci used by Lin et al.^22^ to reconstruct a phylogeny of Limacodidae. We supplemented these data with corresponding data retrieved from our transcriptomes of *C. monomorpha* and *A. stimulea*, and estimated a phylogeny by maximum likelihood (Fig. 3). In this phylogeny, *C. monomorpha* is sister species to *Anaxidia lozogramma*, and a member of a clade of Australian species that also includes *D. vulnerans*, within lineage 6 of the phylogeny of Lin et al.^22^. Consistent with previous studies^23^, the genus *Comana* was not monophyletic. The sister clade to the Australian lineage 6 is the *Parasa* complex, which includes the saddleback caterpillar *Acharia stimulea*.

### *Comana monomorpha* venom contains aerolysin-like proteins

**Figure 4:**
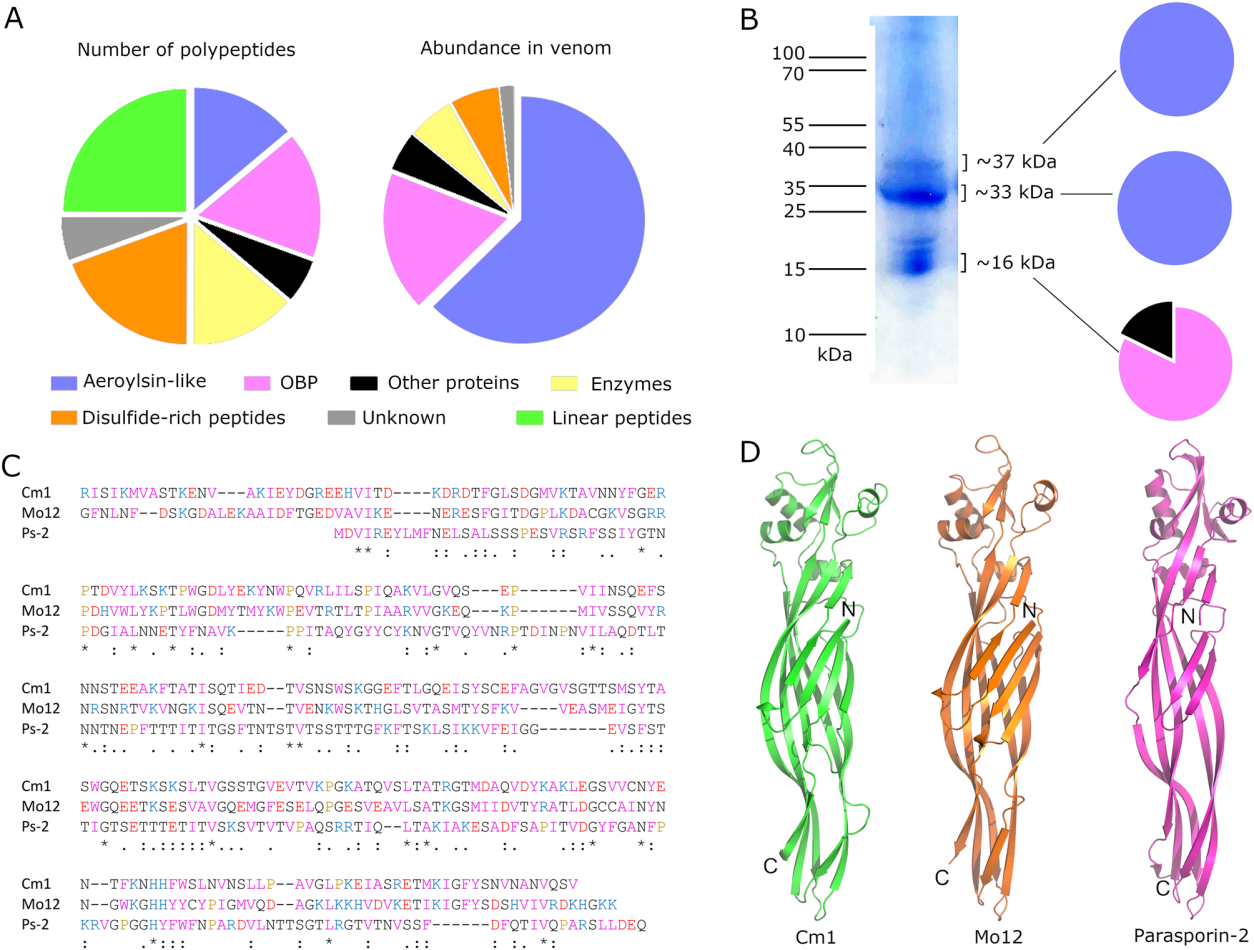
Proteome of *Comana monomorpha* venom. (**A**) Left, number of polypeptide precursors detected using LC–MS/MS; right, protein abundance according to normalised spectral counting. OBP, odorant binding protein. (**B**) SDS-PAGE gel of 40 µg venom. Pie charts on right show protein abundance in each of three gel bands analysed using LC–MS/MS; legend as in panel A. (**C**) Alignment of aerolysin-like proteins from *C. monomorpha* (Cm1), *Megalopyge opercularis* (Mo12), and the bacterial PFP parasporin-2 (Ps-2). (**D**) Comparison of Alphafold 2 predicted structures of Cm1 (green) and Mo12 (orange) with the crystal structure of monomeric form of parasporin-2 (pink, PDB 2ztb).

To investigate the proteomic composition of *C. monomorpha* venom, we combined transcriptomics of venom scoli and proteomics of venom (Fig. 4, Supplementary Data S1). From the reduced, alkylated, and trypsinised (RAT) sample, a total of 28 polypeptide precursors were detected, and 8 additional intact mature peptides were detected from the reduced and alkylated (RA) venom sample. These 36 polypeptides comprise aerolysin-like proteins, odorant binding proteins (OBPs), enzymes, linear and disulfide-rich peptides (Fig. 4A, left). The most abundant toxin family detected in the venom were the aerolysin-like proteins, which make up an estimated 62% of the venom according to MS spectral counts, followed by odorant-binding proteins (18%), disulfide-rich peptides (6%) and enzymes (6%) (Fig. 4A, right). Gel electrophoresis of *C. monomorpha* venom resolved three bands (Fig. 4B). The most intense two bands occur between 30 and 40 kDa and were identified using LC–MS/MS as consisting of multiple aerolysin-like proteins. The band at ~ 16 kDa consisted mostly of proteins of the OBP family.

The aerolysin-like venom proteins of *C. monomorpha* are most similar to those produced by megalopygid caterpillars. For example, the abundant venom protein U-LCTX_66_-Cm1 has 34% identity and 54% similarity to the megalopygid venom protein U-LCTX_8_-Mo12, and lesser identity (17%) and similarity (33%) to bacterial PFPs such as parasporin-2 (Fig. 4C). The Alphafold 2 predicted structure of Cm1 has a predicted local distance difference test (pLDDT) score of 92.4% and is highly similar to the predicted structure of Mo12 and the crystal structure of the monomeric form of parasporin-2 (PDB 2ztb, Fig. 4D).

We detected nine linear and seven disulfide-rich peptides (polypeptides with the mature form containing ≤ 100 amino acid residues) in venom of *C. monomorpha*, including peptides similar to single domain von Willebrand factor peptides, low-density lipoprotein domain peptides, inhibitor cystine knots, and cystine-stabilised α/β-defensins (Supplementary Figure S2). While these peptides represent 44% of precursor sequences detected in the venom, they account for only an estimated 6.4% of spectral counts detected from venom (Fig. 4A). Of the three abundant peptide families previously reported in limacodid venom, ACP-like peptides and inhibitor cystine knots are present as minor components, but cecropin-like peptides are absent. We note that quantification of short peptides may not be accurately measured using spectral counting methods such as those reported here. Nevertheless, our combined datasets suggest that *C. monomorpha* venom contains low levels of peptides in comparison to aerolysin-like proteins.

We conclude that venom of *C. monomorpha* consists mostly of aerolysin-like proteins, a markedly different composition to the peptide-rich venoms produced by the limacodid species previously examined, i.e. *D. vulnerans* and *A. stimulea*^3,4^. The venom composition in *C. monomorpha* is instead more similar to venom produced by megalopygid caterpillars such as *M. opercularis*, which contain multiple aerolysin-like proteins called megalysins^5^.

### *C. monomorpha* venom potently permeabilises mammalian cells

**Figure 5:**
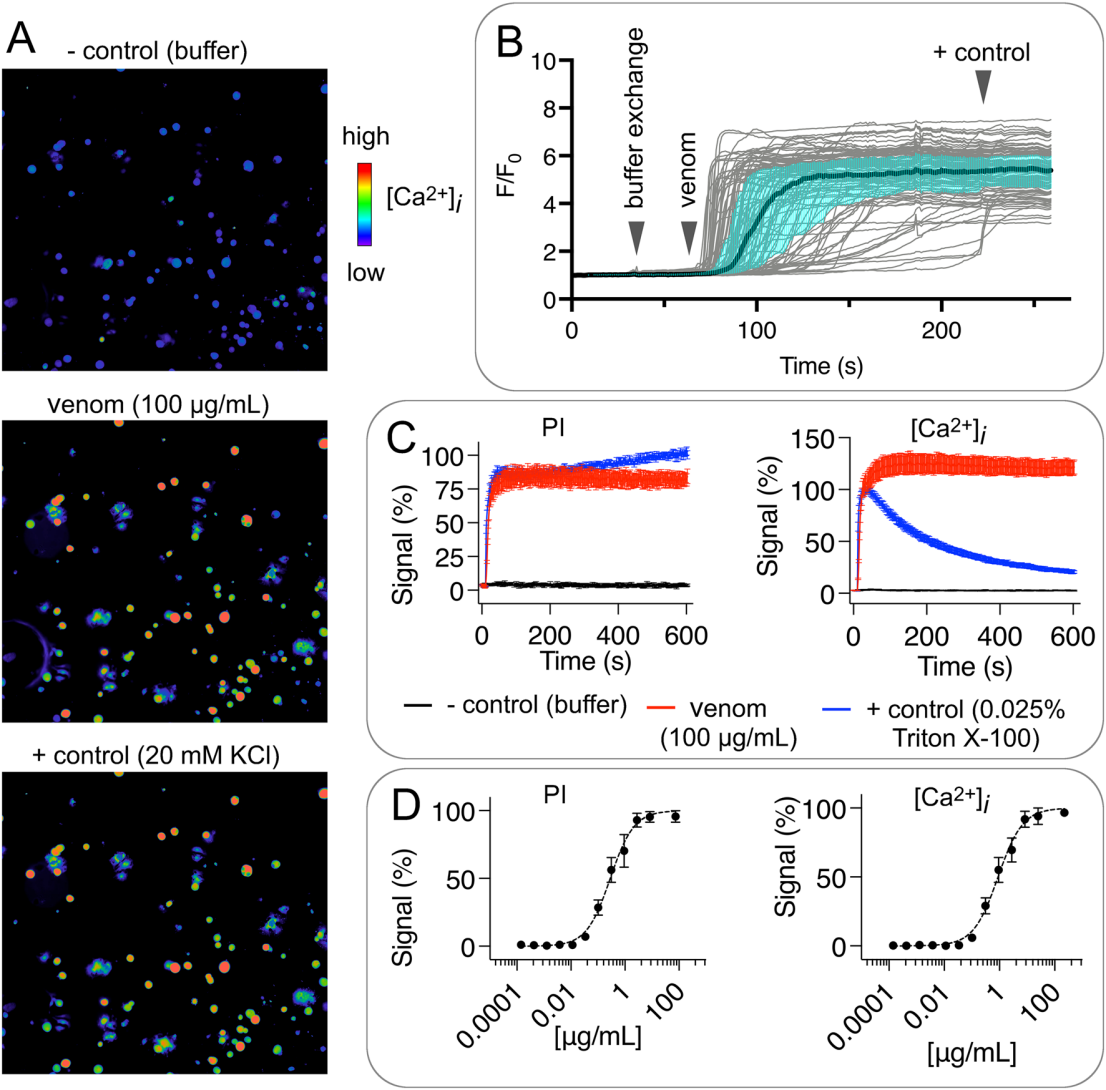
Pore-forming proteins in *Comana monomorpha* venom. (**A**) Ca^2+^ influx into DRG cells after exposure to venom (100 μg/mL). Panels on the left show cells before and 20 s after venom application, and after application of positive control (20 mM KCl). The panel on the right shows fluorescence increase for individual cells (grey lines), median and interquartile ranges (light blue). (**B**) Venom-induced permeabilisation of SH-SY5Y cells as visualised by propidium iodide (PI) access to nucleic acids. Red, *C. monomorpha* venom (100 μg/mL); blue, Triton 0.025%; black, buffer control. (**C**) Ca^2+^ influx into SH-SY5Y cells induced by venom, colours as in panel B. (**D**) Concentration-dependent permeabilisation measured by PI fluorescence (EC_50_ = 284 ng/mL). Each data point shows the average ± SD of three technical replicates, while the curves are four-parameter nonlinear regressions. (**E**) Concentration-dependent Ca^2+^ influx (EC_50_ = 888 ng/mL).

To test the bioactivity of whole venom *of C. monomorpha*, we applied venom (100 µg/mL) to cultured mouse peripheral sensory neurons and human neuroblastoma cells (Fig. 5). Venom caused a strong and sustained Ca^2+^ influx into DRGs, an indicator of sensory neuron activation associated with nociception^24^. SH-SY5Y cells also responded to 100 µg/mL venom with a strong Ca^2+^ influx and propidium iodide (PI) signal resulting from permeabilisation of the cell membrane. Exposure to a dilution series of venom revealed potencies (EC_50_) of 888 ng/mL (95% confidence interval, CI, 715–1106 ng/mL) and 284 ng/mL (95% CI 222–364 ng/mL) for Ca^2+^ influx and permeability to PI, respectively. The high potency and sustained, rather than transient, Ca^2+^ and PI signals in this assay are more similar to our previously observed activity of megalopygid venoms^5^. These results are consistent with the presence of PFPs in the venom and the ability of venom to cause the pain and tissue damage observed after electric caterpillar envenomation.

## Discussion

Australia’s electric caterpillar *C. monomorpha* is a unique species with respect to our current knowledge: it is endemic to a small region of coastal north-eastern Australia, protected by a dense covering of venomous spines, and produces an envenomation syndrome characterised by longer-lasting pain and more visible tissue damage compared to other limacodids such as *D. vulnerans*. On the basis of these observations, we hypothesised that *C. monomorpha* has a different venom composition from *D. vulnerans*.

We found that venom of *C. monomorpha* is rich in aerolysin-like PFPs. PFPs form the largest class of bacterial protein toxins^25,26^. They have been found in both Gram-positive and Gram-negative bacteria and include many distinct protein families such as AB and ABC toxins, membrane attack complex/perforin/cholesterol-dependent cytolysins, haemolysins, and the aerolysin/epsilon toxin/mosquitocidal protein family^27–29^. PFPs occur across the tree of life, such as amaranthin-like proteins in plants^30^ and aerolysin-like proteins in vertebrates^31^, likely due to both vertical and horizontal gene transfer^32^. Aerolysin family proteins have been recruited as venom toxins in many animal groups, including the natterins found in fish venoms, β-pore forming toxins (β-PFTx) in centipede venoms, and actinoporins in Cnidaria^32–34^. Recently, we reported that the venom of asp caterpillars (*Megalopyge* spp.) also contains PFPs^5^.

The marked differences in venom production and composition between *D. vulnerans* and *Megalopyge* spp. previously led us to propose that venom has arisen separately in the Megalopygidae and Limacodidae^6^ despite their close relationship within Zygaenoidea^35^. However, the similarities between the venom of the limacodid *C. monomorpha* and megalopygid caterpillars we observed in this study may instead suggest a common origin of venom. However, the evolution of venom composition within Zygaenoidea is not yet clear from current data: *D. vulnerans* and *C. monomorpha* are more closely related to each other than either is to *A. stimulea*, and all three species are only distantly related to *M. opercularis*. Despite this, venoms of *C. monomorpha* and *M. opercularis* are dominated by aerolysin-rich venoms, whereas the venoms of *D. vulnerans* and *A. stimulea* are peptide-rich. Examination of the venom composition of additional zygaenoid species with a broader phylogenetic spread is required to delineate the pathway of venom evolution in Zygaenoidea. A detailed analysis of the phylogeny of aerolysin-like venom toxins produced by Limacodidae and Megalopygidae would also provide insights.

Venom of *C. monomorpha* causes pain and tissue damage, and its principal molecular mode of action is permeabilisation of cell membranes. The bioactivity of electric caterpillar venom is different from that of limacodids such as *D. vulnerans*, which also disrupts cell membranes to cause pain, but which relies on cecropin-like peptides to do so^4^. In vitro, venom of *D. vulnerans* and synthetic cecropin-like peptides have been shown to cause rapid but transient activation of sensory neurons, whereas the venom produced by *C. monomorpha* and the megalopygid *M. opercularis* cause a more sustained response, consistent with distinct modes of membrane permeabilisation. In vivo, *D. vulnerans* cecropin-like peptides cause immediate but short-lasting pain in mice^4^, whereas asp caterpillar venom (*Megalopyge* spp.) causes pain that persists for more than 30 min^5^. Anecdotal evidence (including the authors’ observations) indicates that in humans, envenomations by *D. vulnerans* cause short-lasting pain (similar to a honeybee sting), whereas those of *C. monomorpha* persist much longer. Likewise, we are not aware of stings by *D. vulnerans* causing long-lasting lesions or anaphylaxis-like symptoms as reported by victims of envenomation by *C. monomorpha*. We hypothesise that the cecropin-like toxins are associated with short-lasting pain *D. vulnerans* envenomation, while the pore-forming proteins in *C. monomorpha* and megalopygid venoms are associated with longer-lasting and more severe symptoms.

This study provides a species identity for the electric caterpillar, a documented case study of envenomation, and reveals its venom composition and bioactivity. In addition, it highlights the diversity of zygaenoid venoms, and the need to study a broader range of species to fully understand the trajectory of venom evolution in Zygaenoidea.

### Supplementary Information

Supplementary Information 1.

Supplementary Figures.

## Data Availability

Raw sequencing reads used in this project are available in fastq format from the National Center for Biotechnology Information (NCBI) Sequence Read Archive (BioProject PRJNA1086036) and the assembled contigs from the Transcriptome Shotgun Assembly database (accession number GKSQ00000000). MS data have been deposited to ProteomeXchange consortium^36^ (PRIDE ID PXD051063). The final proteome, comprising 36 amino acid sequences and the ORFs encoding them, was submitted to NCBI and assigned GenBank accession numbers PP480802–PP480837.
