# Supplementary material for: Phylogeny, envenomation syndrome, and membrane permeabilising venom produced by Australia’s electric caterpillar Comana monomorpha
Source: Sci Rep. 2024 Jun 19;14:14172. doi: 10.1038/s41598-024-65078-1 (PMC11187147; doi:10.1038/s41598-024-65078-1)
Supplement: Supplementary file 2 — Supplementary Figures. [file 41598_2024_65078_MOESM2_ESM.pdf]

Supplementary Information

[redacted] 30 Mar 2018 •

Hi All, PLEASE BE WARY WHEN IN YOUR GARDENS - this is the 3rd year in a row that I have been in my garden and come across these caterpillar like things about from March - May each year... The closest identification that I have found is that they could possibly be a mangrove moth caterpillar. They commonly seem to be on the underside of the leaves of my rose bushes but I've also just found them on my golden cane palms. The problem is that they have the sharp black spikes on either end, so when you brush by them they leave you with a very painful stinging burning sensation, you might think you've brushed by a wasps nest or something so you try to brush them away and end up getting stung on your hands too... Your skin automatically comes up in these hot welts, then [redacted] hours later you get a massive hot, burning, red rash which is sometimes very itchy, [redacted] for up to 7 - 10 days after... It feels like 7 rings of hell...

It's particularly bad for me because my throat and tongue swell up and it becomes hard to breath. I think that may happen anyone, if you have contact with a few at once, it worsens the reaction (like being stung by too many bees is dangerous to people who aren't normally reactive to one bee sting)... I've been to the hospital and been hospitalised over night for this twice. The only thing that helps is antihistamines, which I take the moment o come into contact with them now...

Please also let me know if you've experienced anything like this or have them in your area also. I'm interested to know where to people have spotted them, what types of plants they are occurring on... I'm located in Annandale near Annandale Central and I have them on the my rose bushes and golden canes and they are usually on the underside of the leaf.

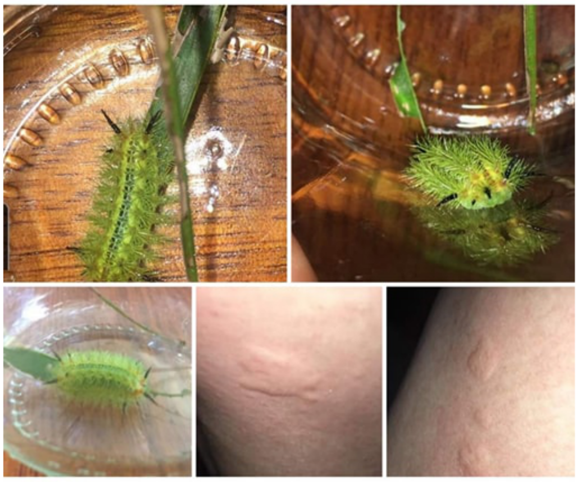

[redacted] and 13 others 16 Comments

Like Comment

Related keywords

[redacted] [redacted]

[redacted]

Its that time of year again... Just got stung between my toes by one of these 😡... Fortunately I dug out my prednisone [redacted] quickly, so heres hoping i dont get the excruciating pain again for the next week [redacted]

Supplementary Figure S1: Anecdotal evidence from social media of electric caterpillar envenomation causing long-lasting and anaphylaxis-like effects.

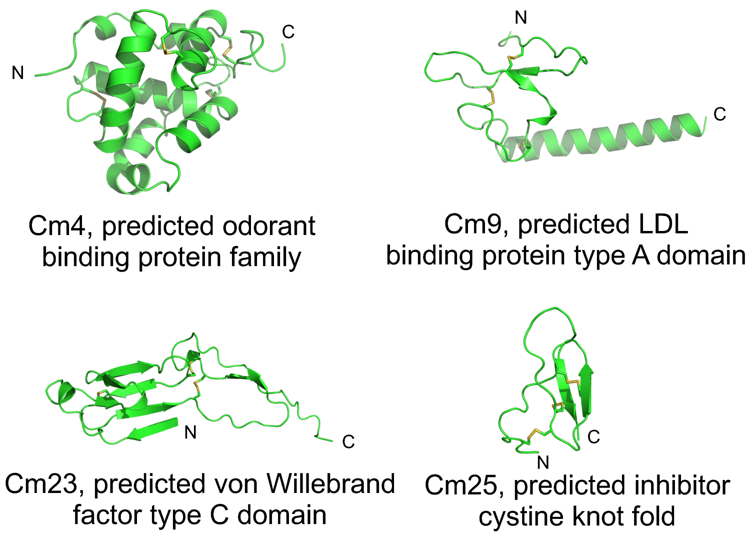

Supplementary Figure S2: Predicted folds of selected peptides and proteins in electric caterpillar venom. LDL, low density lipoprotein. Alphafold 2 pLDDT scores for these structures are 85.2 (Cm4), 85.1 (Cm9), 88.1 (Cm23), and 65.8 (Cm25).

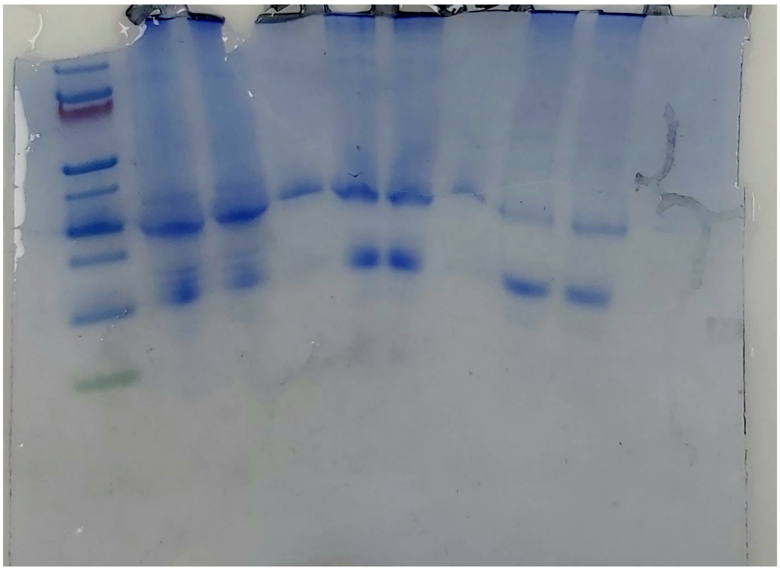

Figure S3: Raw gel image from which the cropped image in Figure 4B was obtained.

'

Supplementary Data S1: Annotated venom proteome of *C. monomorpha*.
